# Extracellular vesicle-mediated chemoresistance in breast cancer: focus on miRNA cargo

**DOI:** 10.20517/evcna.2024.90

**Published:** 2025-02-24

**Authors:** Maria Chiara Ciferri, Roberta Tasso

**Affiliations:** ^1^Department of Experimental Medicine (DIMES), University of Genova, Genova 16132, Italy.; ^2^Dipartimento della Ricerca, IRCCS Ospedale Policlinico San Martino, Genova 16132, Italy.

**Keywords:** Breast cancer, chemotherapy, circulating EVs, drug resistance

## Abstract

The role of extracellular vesicles (EVs) in mediating chemoresistance has gained significant attention due to their ability to transfer bioactive molecules between drug-resistant and drug-sensitive cells. In particular, they have been demonstrated to play an active part in breast cancer chemoresistance by the horizontal transfer of genetic and protein material. This review highlights the role of EVs, particularly their miRNA cargo, in driving drug resistance in breast cancer. EVs derived from chemoresistant cells carry miRNAs and lncRNAs, which are known to modulate gene networks involved in cell proliferation and survival. These cargo molecules suppress apoptosis by targeting pro-apoptotic genes like PTEN and BIM, promote epithelial-mesenchymal transition (EMT) through the regulation of pathways such as TGF-β and Wnt/b-catenin, and contribute to tumor growth and resistance by enhancing angiogenesis and modulating the tumor microenvironment. Beyond RNA-mediated effects, EVs also transfer functional proteins, including P-glycoprotein and Hsp70, which impact cellular metabolism and survival pathways. Our findings underscore the significance of EVs in breast cancer chemoresistance, suggesting their potential involvement as possible prognostic factors to predict therapy response and as therapeutic targets in combination with usual therapy.

## INTRODUCTION

### Epidemiology and therapeutic approaches in breast cancer

Cancer remains a significant global health challenge, with over 19.3 million new cases and nearly 10 million deaths reported worldwide in 2024^[1]^. In particular, breast cancer (BC) stands as the most prevalent cancer in women, contributing to approximately 11.7% of all new cancer cases^[1]^. Despite substantial progress in early detection, such as advanced imaging techniques and biomarker-based screening, as well as the development of innovative treatment modalities, survival rates continue to vary, largely depending on the stage of diagnosis and the intrinsic biological characteristics of the tumor^[2]^. These disparities underscore the pressing need for effective prognostic biomarkers that can stratify patients based on their risk and guide personalized therapeutic approaches. Modern cancer therapies, encompassing surgery, radiation, chemotherapy, immunotherapy, and targeted therapies, have markedly improved patient outcomes^[3]^. Immunotherapy has revolutionized cancer treatment by harnessing the immune system and targeted therapies have enabled precise interventions based on tumor-specific molecular aberrations^[4]^. However, the clinical landscape remains complicated by therapeutic resistance, tumor heterogeneity, and systemic toxicity, which collectively hamper durable treatment responses^[5]^. Tumor heterogeneity, in particular, presents a major obstacle, as the diverse genetic and phenotypic profiles within and between tumors often lead to treatment failure and disease recurrence^[6]^. Additionally, systemic toxicity associated with conventional therapies significantly^[7]^ impacts patient quality of life, emphasizing the need for more refined therapeutic options^[7]^. Consequently, identifying novel strategies that improve diagnostic accuracy, predict therapeutic outcomes, and enhance treatment specificity is critical. Innovative approaches are now focusing on minimally invasive techniques, such as liquid biopsies, to monitor disease progression and treatment response^[8]^. In this context, efforts to harness the potential of extracellular vesicles (EVs) and other circulating biomarkers are gaining attention, offering promising avenues for the early detection of cancer, monitoring metastatic progression, and overcoming therapy resistance^[9]^. Although still at an early stage and hampered by significant challenges in the standardization of procedures for detecting and characterizing circulating EVs, this field holds promise for advancing oncology by potentially enabling more personalized and precise diagnostic approaches.

### Extracellular Vesicles as information carriers and their role in cancer

The term extracellular vesicle refers to lipid bilayer-enclosed nano- to micro-sized particles that cannot replicate independently and are secreted from all cell types^[10]^. Being able to exchange proteins, lipids, and genetic material, EVs can be considered key players in intercellular communication^[11]^. Based on their biogenesis, they can generally be classified into exosomes and ectosomes. Exosomes originate from the cell's internal compartments (endosomal system) and are released via multivesicular body (MVB) formation. Indeed, intraluminal vesicles (ILV) are formed through an inward membrane budding into the endosome lumen (early endosome) which undergoes maturation into MVBs. ILVs are secreted as exosomes when the MVB fuses with the plasma membrane, avoiding lysosomal degradation^[11-13]^. On the other hand, ectosomes (historically known as microvesicles) bud from outward protrusions of the plasma membrane and shed directly into the extracellular space^[10,13]^. They can arise from large surface blebs, such as those involved in the secretion of large oncosomes, as well as from smaller blebs or cellular protrusions^[13,14]^. Migrasomes, which are vesicles released in a migration mechanism called “migracytosis”^[15]^, and apoptotic bodies, represented by the small fragments produced through the disassembly of apoptotic cells into smaller fragments which are generated via “beads-on-a-string” called “apoptopodia”^[16]^ are also classified as EV subtypes. Exosome formation is heavily reliant on the endosomal sorting complex required for transport (ESCRT) machinery. Nevertheless, ESCRT-independent mechanisms have also been proposed^[11]^. In contrast, ectosome biogenesis involves a combination of ESCRT components, lipid-driven processes, and cytoskeletal rearrangements^[11,17]^.

Different mechanisms have been demonstrated to be involved in the delivery of EV cargo and the subsequent uptake by target cells^[18]^. Specifically, endocytosis, which comprehends lipid-raft-mediated endocytosis and clathrin-/caveolin-mediated endocytosis, phagocytosis, and macropinocytosis, has been historically considered the primary pathway^[19-21]^. The direct fusion of the EV membrane with the cell plasma membrane has been proposed as another possible entryway^[22]^. Additionally, protein interaction can be an essential component of the EV uptake process. In this sense, tetraspanins, integrins, immunoglobulins, proteoglycans, and lectins have been identified as active players in EV internalization^[18]^. Moreover, recent evidence suggests that recipient cells can absorb EV-miRNAs and cause important modulation effects, not only in a pathogenic context but especially in physiological processes^[23-25]^.

In 2006, Ratajczak *et al*. observed that embryonic stem cell (ESC)-derived vesicles were able to enhance the survival and expansion of hematopoietic progenitor cells, and upregulate their expression of early pluripotent and hematopoietic stem cell markers^[26]^. Given previous evidence of mRNA enrichment in apoptotic bodies^[27,28]^, they observed that ESC-EVs were highly enriched in mRNAs encoding essential pluripotency-associated transcription factors, which could be transferred to target cells and translated into functional proteins. This was one of the earliest studies demonstrating that EVs contain mRNA and that their horizontal transfer can exert important biological effects^[28]^. Subsequently, further evidence that exosomes contain functional mRNAs and microRNAs (miRNAs) was demonstrated, defining this EV-mediated process as a novel mechanism of genetic exchange between cells^[29]^. These groundbreaking studies led to extensive research into the role of EVs in genetic material transport and modulation of cellular functions. The EV-mediated delivery of miRNAs, the mechanisms of miRNAs sorting into EVs, and the quantitative aspects of EV uptake by recipient cells are still a topic of ongoing debate within the scientific community. The selective sorting of miRNAs into EVs is a result of multiple mechanisms, as evidenced by recent research. It is regulated by a variety of proteins, particularly those that bind RNA. Over a decade ago, short sequence motifs, termed “EXOmotifs”, were identified as being overexpressed in miRNAs, playing a crucial role in directing their loading into exosomes^[30]^. In addition, altering these motifs can influence the miRNA cargo within EVs. Among the key proteins involved in miRNA regulation, the RNA-binding protein heterogeneous nuclear ribonucleoprotein A2B1 (hnRNPA2B1) has been shown to control miRNA loading into EVs by binding to miRNAs through their EXOmotifs. The sumoylation of hnRNPA2B1 has been identified as a critical post-translational modification controlling hnRNPA2B1–miRNA binding^[30]^. A more recent study further demonstrated the active role of hnRNPA2B1 in sorting mRNAs and lncRNAs into EVs^[31]^. The gap junction protein Connexin43 (Cx43) was found to modulate the selective incorporation of specific miRNAs into EVs, either through direct binding or in association with RNA-binding proteins, such as hnRNPA2B1 and hnRNPQ. Moreover, Cx43 was shown to facilitate EV-miRNA delivery into recipient cells^[32]^. HnRP-Q, also known as synaptotagmin-binding cytoplasmic RNA-interacting protein (SYNCRIP), was also identified as a key component of the hepatocyte exosomal machinery controlling miRNA sorting^[33]^.

Recent research also explored how different EV subpopulations with distinct physical characteristics may be linked to different molecular mechanisms of miRNA sorting. A study on MDA-MB 231-derived small EVs (sEVs) identified two subpopulations with different densities, each associated with distinct miRNA sorting strategies^[34]^. Specifically, one subpopulation exhibited non-selective miRNA packaging, while the other displayed both non-selective and selective packaging mechanisms. The RNA-binding protein Lupus La has been identified as a mediator in the sorting of selectively packaged miRNAs, especially for miR-122, which was found to contain two motifs responsible for high-affinity binding to Lupus La and subsequent sorting into vesicles in a cell-free system^[34]^. Another important RNA-binding protein involved in this sorting process is Y-box protein 1 (YBX1), which plays a role in sorting miRNAs and other non-coding RNAs into EVs^[35]^. Quantifying miRNA-rich EVs within a heterogeneous EV population is challenging, especially when relying on density-based ultracentrifugation for isolation. For instance, in a study using murine bronchoalveolar lavage fluids, miRNA-rich EVs represented only 6% of the total EV population^[36]^. Moreover, 10% of total EVs isolated from conditioned media of colon cancer cells were found to be miRNA-rich EVs^[37]^. This was consistent with stoichiometric calculations showing that miRNA content in highly purified EV preparations was very low, with less than one copy/EV for all the analyzed miRNAs^[38]^.

The role of EVs is particularly relevant in cancer biology, where particles originating from tumors can modulate the surrounding tissue environment and impact the behavior of both malignant and non-malignant cells^[39]^. In breast cancer, tumor-derived EVs have gained attention for their ability to convey molecular signals that drive disease progression, metastasis, and treatment resistance^[40]^. Circulating EVs, enriched in bioactive molecules, act as "molecular messages" that orchestrate a range of cellular responses in recipient cells^[41]^. For example, EVs can facilitate the transfer of oncogenic signals, including altered gene expression patterns, immune modulation factors, and metabolic reprogramming signals^[42,43]^. These activities highlight EVs as essential players in the systemic dissemination of cancer-related signals. Notably, EVs can contain chemokines and cytokines that recruit immune cells, potentially promoting an immunosuppressive microenvironment that enables cancer cells to evade immune detection^[44]^. Additionally, the lipid composition of EV membranes can play a role in cell signaling processes, contributing to cancer cell survival by protecting transported bioactive molecules from enzymatic degradation and enhancing EV fusion with recipient cells^[45,46]^. This lipid composition, often enriched with phosphatidylserine and other membrane lipids, facilitates EV uptake by recipient cells through receptor-mediated endocytosis^[47]^. Further, the membrane proteins on EVs, such as integrins and tetraspanins, can influence specific tissue tropism, allowing EVs to preferentially interact with and deliver cargo to certain cell types, including those at pre-metastatic niches^[48-52]^. Together, these features position circulating EVs as critical modulators of cellular behavior and environment, actively participating in breast cancer progression and metastasis. Beyond their ability to carry functional molecules, EVs also promote drug resistance mechanisms by sequestering or transporting therapeutic agents away from their targets^[53]^. For example, EVs can encapsulate cytotoxic drugs, limiting their effective concentration within tumor cells. This ability, combined with the immune-modulatory functions of EVs, allows them to reshape the tumor microenvironment in favor of cancer survival and spread^[54]^. Through their capacity to alter cellular pathways at multiple levels, circulating EVs have emerged as key facilitators in the progression of breast cancer and other malignancies, holding potential as biomarkers and therapeutic targets.

**Figure 1:**
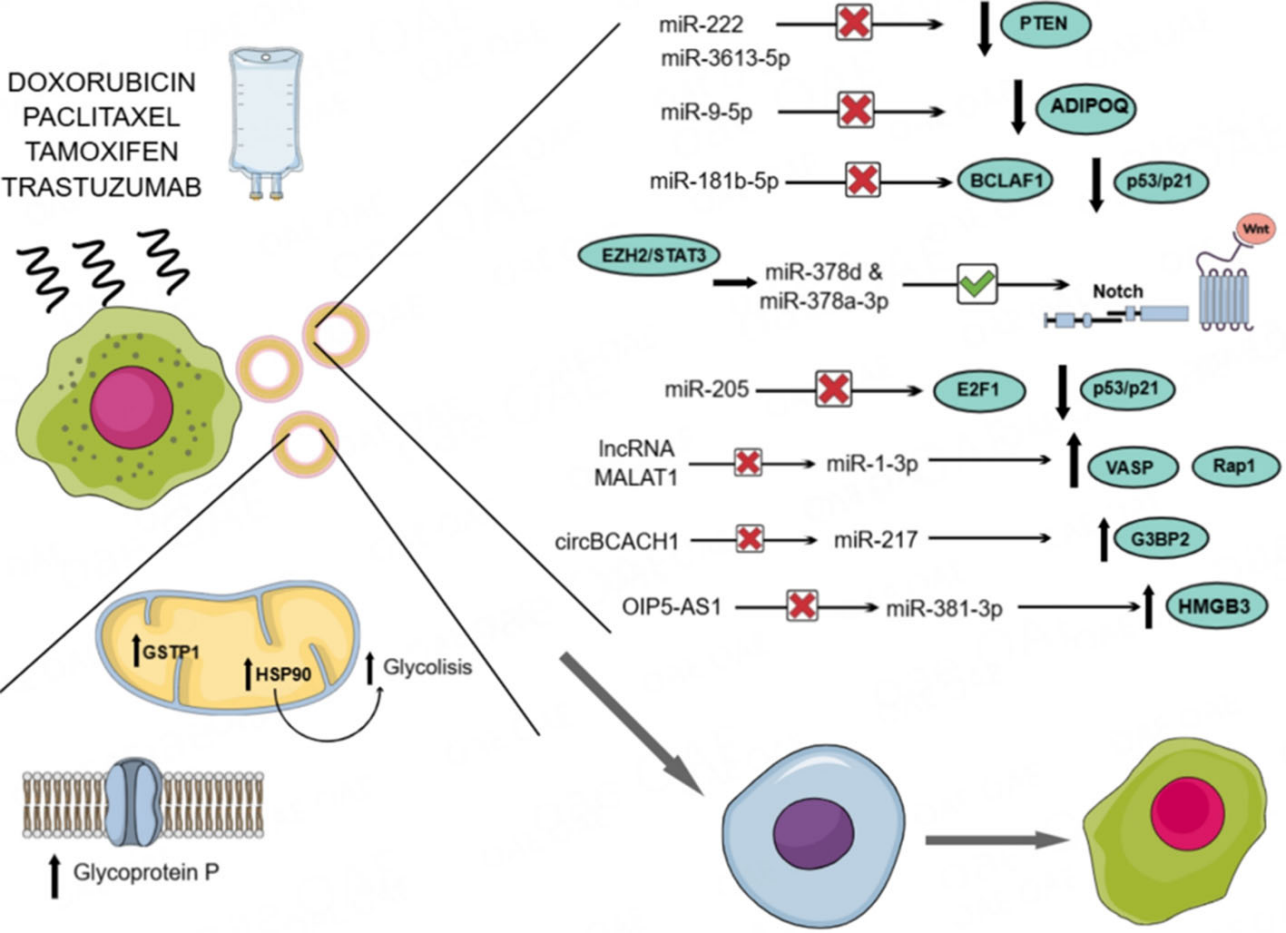
Main molecular pathways involved in chemoresistance spreading related to EV-cargo. EV: extracellular vesicle.

Several recent studies have highlighted extracellular vesicles' crucial role in chemotherapy resistance mechanisms^[55-63]^. This review aims to summarize and discuss the research dedicated to evaluating these processes in the context of breast cancer [Figure 1 and Table 1]. Most studies in this area have identified the genetic cargo of EVs, particularly microRNAs, as the primary contributors to chemoresistance. In contrast, fewer investigations have explored the involvement of other EV-associated molecules. As a result, this work places a stronger emphasis on the findings related to the genetic cargo of EVs. Nevertheless, some examples of other potential mechanisms have also been included.

**Table 1:** Summary of the different EV cargo responsible for chemoresistance spreading

**Chemotherapy treatment**	**EV cargo**	**Ref**
Doxorubicin	● miR-222 ● miR-181b-5p ● miR-3613-5p ● long non-coding RNA H19 ● long non-coding RNA metastasis-associated lung adenocarcinoma transcript 1 (MALAT1) ● Glutathione S-transferase P1 (GSTP1) ● HSP70	[97] [106] [108] [110] [111] [126] [127]
Tamoxifen	● miR-221/222 ● miR-9-5p ● miR-205	[100] [101] [104]
Doxorubicin + paclitaxel	● miR-155 ● miR-378d & miR-378-3p	[105] [107]
Paclitaxel	circBCACH1	[117]
Trastuzumab	long non-coding RNA OPA-interacting protein 5 antisense transcript 1 (OIP5-AS1)	[119]
Docetaxel	P-glycoprotein (P-gp)	[123]

EV:Extracellular vesicle.

### Mechanisms of chemoresistance in breast cancer

Chemoresistance in breast cancer (BC) is a complex and multifactorial process that allows cancer cells to evade the cytotoxic effects of chemotherapy, thus limiting the effectiveness of conventional treatments^[64]^. Multiple mechanisms drive chemoresistance in BC, and these can generally be grouped into categories that involve: (i) genetic and epigenetic changes, such as DNA methylation and histone acetylation, that can silence tumor suppressor genes or activate oncogenes^[65]^; (ii) cell membrane alterations that influence drug adsorption, transport, and efflux. Indeed, overexpression of efflux transporters, such as P-glycoprotein (P-gp) and Breast Cancer Resistance Protein (BCRP), reduces intracellular drug concentrations and diminishes chemotherapy efficacy^[66,67]^; (iii) enhanced DNA repair capabilities^[68]^; (iv) changes in the tumor microenvironment, driven, for example, by cancer-associated fibroblasts (CAFs) and immune cells that secrete factors that protect tumor cells from chemotherapy-induced apoptosis and promote stem-like phenotypes^[69]^, and (v) metabolic reprogramming. Chemoresistant BC cells frequently exhibit a shift toward glycolysis (Warburg effect) and altered lipid metabolism to sustain growth and survival under therapeutic stress^[70]^.

As stated above, one primary mechanism of chemoresistance is the upregulation of drug efflux transporters^[71]^. Transporters in the ATP-binding cassette (ABC) family, such as P-glycoprotein (P-gp), breast cancer resistance protein (BCRP), and multidrug resistance protein 1 (MRP1), actively pump chemotherapeutic agents out of cancer cells, reducing intracellular drug concentration and lowering drug efficacy^[72]^. Overexpression of these efflux pumps has been observed in many chemoresistant breast cancer cell lines and is often associated with cross-resistance to multiple drugs^[73,74]^. MiRNAs further contribute to this process by regulating the expression of these transporters^[75,76]^. For instance, downregulation of miR-451 has been shown to increase the expression of the ABC transporter ABCB1, promoting drug efflux and chemoresistance^[77]^. Conversely, overexpression of miR-326 has been associated with reduced levels of BCRP, which sensitizes cells to chemotherapeutic agents^[76]^. The ability of miRNAs to fine-tune the expression of drug transporters represents a critical pathway enabling breast cancer cells to develop resistance.

Another crucial mechanism through which miRNAs mediate chemoresistance involves the modulation of apoptotic pathways^[78]^. MiR-21, one of the most commonly upregulated miRNAs in breast cancer, targets and downregulates the expression of tumor suppressor genes such as PTEN, PDCD4, and BCL-2, which are involved in cell cycle regulation and apoptosis^[79]^. By inhibiting pro-apoptotic factors and promoting anti-apoptotic pathways, miR-21 allows cancer cells to survive despite exposure to chemotherapeutic agents, contributing to resistance. Additionally, miR-221 and miR-222 have been shown to downregulate P27KIP1 and TIMP3, which are key regulators of apoptosis, further enhancing breast cancer cell survival under chemotherapeutic stress^[80]^. Epigenetic modifications, such as DNA methylation, histone alterations, and regulation by non-coding RNAs, are also key contributors to chemoresistance in breast cancer^[81]^. These changes can either silence genes that enhance chemosensitivity or activate those that promote drug resistance. For instance, the hypermethylation of promoter regions in tumor suppressor genes can reduce their expression, enabling cancer cells to evade the effects of chemotherapy. Additionally, specific modifications to histones can reshape chromatin structure, limiting the accessibility of DNA to drugs that target nuclear components, further diminishing the efficacy of treatment^[82]^.

Enhanced DNA repair capabilities play a significant role in conferring resistance to chemotherapy^[83]^. Indeed, upregulation of the DNA repair pathways, such as homologous recombination and nucleotide excision repair, enables cancer cells to tolerate DNA-damaging agents like platinum-based drugs^[84]^. Inhibitors of DNA repair pathways, such as PARP (poly ADP-ribose polymerase) inhibitors, have been explored as therapeutic agents to target chemoresistant BC cells with DNA repair deficiencies^[85]^. MiRNAs further reinforce this resistance by modulating key genes involved in DNA repair mechanisms. For example, miR-155, which is upregulated in chemoresistant BC, enhances the expression of genes involved in homologous recombination and non-homologous end joining, such as *RAD51* and *DNA-PK*, thereby improving the cells' ability to repair chemotherapy-induced DNA damage^[86]^. Similarly, miR-182 targets *BRCA1*, a crucial gene in the DNA damage response pathway, and its overexpression has been linked to defective DNA repair, resulting in increased resistance to DNA-damaging agents commonly used in chemotherapy^[87]^. These miRNA-driven effects on DNA repair pathways further contribute to the development of chemoresistance in breast cancer.

The tumor microenvironment (TME) also plays a critical role in chemoresistance^[88]^. The TME consists of various non-cancerous cells, such as fibroblasts, immune and endothelial cells, and the extracellular matrix, all of which interact dynamically with cancer cells^[89]^. These interactions can promote chemoresistance in multiple ways. For example, cancer-associated fibroblasts (CAFs) can secrete cytokines and growth factors that enhance the survival and proliferation of BC cells. MiRNAs such as those belonging to the miR-200 family also play a critical role in TME-mediated resistance by regulating epithelial-to-mesenchymal transition (EMT), a process that increases cell invasiveness and chemoresistance. MiR-200b and miR-200c, for example, target transcription factors like ZEB1 and ZEB2, which are central to EMT^[90]^.

Finally, metabolic reprogramming, another hallmark of cancer, is also regulated by miRNAs and plays a role in chemoresistance^[91]^. MiR-34a, for example, is downregulated in chemoresistant BC cells and is involved in the regulation of glucose metabolism. Reduced levels of miR-34a have been reported to increase the expression of LDHA, an enzyme that promotes glycolysis, which supports the metabolic needs of cancer cells and enables them to survive under chemotherapy-induced stress^[92]^. Additionally, miR-122 has been implicated in modulating fatty acid metabolism. Inhibition of miR-122 leads to enhanced lipid synthesis and storage, providing an energy reserve that promotes cell survival during treatment^[93]^.

In summary, chemoresistance in breast cancer arises through a multifaceted array of mechanisms. Understanding these pathways is essential for developing more effective therapeutic strategies to overcome chemoresistance and improve patient outcomes.

## THE ROLE OF EXTRACELLULAR VESICLES (EVs) IN BREAST CANCER CHEMORESISTANCE

### Horizontal transfer of genetic material: EV-associated miRNA transfer

It is well established that EVs carry nucleic acids that function as signaling molecules and play a pivotal role in various diseases^[29,94]^. Notably, EVs have been described to be active contributors to tumor development and progression, particularly in breast cancer^[95,96]^. In recent years, research has increasingly focused on whether the nucleic acids within the EV cargo are implicated in therapy resistance. Early studies primarily aimed to conduct a broad analysis to demonstrate that EVs derived from drug-resistant cells could transfer chemoresistance to drug-sensitive cells and possess a distinct miRNA profile compared to EVs derived from corresponding drug-sensitive cells. This phenomenon was initially demonstrated through in vitro co-culture experiments using MCF-7 cells resistant to either adriamycin or docetaxel and with their corresponding drug-sensitive parental cells. A resistance transmission mechanism was observed in sensitive cells upon co-culture with resistant ones, resulting in significantly increased survival rates. Importantly, this effect was partially ascribed to EVs released by resistant cells^[97]^. MiRNA profiling of EVs derived from resistant cells was conducted and compared to the levels observed in sensitive cells after stimulation with the same EVs. The profiles revealed selective miRNA patterns, with enrichment in miR-100, miR-222, miR-30a, and miR-17, which were also significantly increased in the recipient-sensitive cells. Pathway prediction analysis indicated that miR-100 and miR-222 were associated with “pathways in cancer”, “cell cycle”, and “MAPK signaling pathway”, all of which are well-known contributors to various tumor-related cellular processes, including chemoresistance^[98]^. Additionally, the relative expression of selected miRNAs in MCF-7 sensitive cells after treatment with EVs released by resistant cells revealed significant downregulation of PTEN, a tumor suppressor that regulates multidrug resistance in breast cancer, targeted by miR-222^[97]^. PTEN’s role in modulating multidrug resistance in breast cancer is well-documented^[99]^.

The role of EV-associated miRNA-221/222 in mediating chemoresistance has also been explored in another study, highlighting its contribution to tamoxifen resistance in BC cells^[100]^. This work demonstrated that EVs derived from tamoxifen-resistant MCF-7 cells differed significantly in size, protein composition, RNA content, and overall quantity compared to EVs from tamoxifen-sensitive ones. Furthermore, it was shown that these EVs could partially transfer tamoxifen resistance to sensitive cells through the delivery of miRNA 221/222. Notably, this effect was effectively inhibited by the use of an anti-miRNA-221/222, underscoring the pivotal role of these miRNAs in the observed resistance mechanism^[100]^. Another EV-associated miRNA was identified as potentially responsible for tamoxifen resistance^[101]^. EVs isolated from both tamoxifen-resistant and -sensitive MCF-7 cells were tested in vitro and in vivo for their ability to enhance drug resistance, confirming the hypothesis in both cases. Through bioinformatic analysis, differentially expressed genes in EVs derived from resistant cells were identified, including the ADIPOQ gene and its regulatory miRNA, miR-9-5p. Further investigation revealed that miR-9-5p downregulated ADIPOQ expression. The enrichment of miR-9-5p in EVs could explain its role in transmitting drug resistance, supported by prior studies linking high miR-9-5p expression in primary breast tumors to advanced disease and metastasis^[102]^, while increased ADIPOQ expression was associated with improved survival outcomes in chemotherapy-treated BC patients^[103]^.

Another noteworthy study highlighted the role of EV-associated miRNA-205 in tamoxifen resistance and breast cancer progression^[104]^. The study analyzed BC cells isolated from tumor tissues of two patients who had undergone surgical resection. MiRNA-205 was found to be upregulated in tamoxifen-resistant MCF-7 cells and their EVs. When these EVs were internalized by BC cells, they promoted drug resistance, proliferation, migration, and invasion, while suppressing apoptosis. Similar effects were observed in vivo after intratumoral injection of these EVs.

Following this, other EV-associated miRNAs were recognized as key players in drug resistance. In 2018, Carvalho Santos *et al*.^[105]^ reported that cancer cells receiving miRNA-155-enriched EVs exhibited significantly higher resistance to doxorubicin and paclitaxel compared to control cells, suggesting miRNA-155 as a potential mediator of drug resistance transmission.

Another potential mechanism of EV-mediated drug resistance, specifically to doxorubicin (DOX), was proposed in a study that identified the role of EV-associated miR-181b-5p^[106]^. Using small-RNA sequencing and bioinformatic analysis, researchers found that miR-181b-5p was enriched in EVs derived from drug-resistant BC cells. The transfer of these EVs to sensitive cells was linked to increased DOX resistance. Mechanistically, miR-181b-5p was shown to suppress cellular senescence and promote the resistant phenotype by targeting BCLAF1, leading to a downregulation of the p53/p21 pathway. Additionally, analysis of EVs from the serum of 24 patients before and after neoadjuvant chemotherapy (NAC) revealed that elevated levels of EV-associated miR-181b-5p correlated with poor chemotherapy response, highlighting its clinical relevance as a potential biomarker and therapeutic target.

Further evidence of EV-mediated chemoresistance was provided by a clinical study that analyzed EVs isolated from patients before receiving chemotherapy (doxorubicin and paclitaxel), after one and after four cycles^[107]^. MiRNA sequencing revealed that miR-378a-3p and miR-378d levels were significantly elevated following therapy, with a more pronounced increase in patients who were chemotherapy-insensitive. These findings highlighted a novel mechanism of acquired resistance involving the EZH2/STAT3 axis. Specifically, STAT3 was shown to bind to the promoter regions of miR-378a-3p and miR-378d, driving their enrichment in EVs. Upon internalization by neighboring cells, these EVs activated the WNT and NOTCH signaling pathways, thereby promoting drug resistance^[107]^.

In addition to these findings, the role of miR-3613-5p as an EV-associated mediator of chemoresistance has also been investigated. Luo *et al*.^[108]^ reported a significant upregulation of miR-3613-5p in DOX-resistant BC tissues and cells. Using an RNase A protection assay, they demonstrated that miR-3613-5p was encapsulated within EVs as its level was unaffected by treatment with RNase A alone but decreased upon the addition of Triton X-100, confirming its localization inside EVs. Importantly, the EV-mediated transfer of miR-3613-5p to sensitive cells enhanced their resistance to DOX. Mechanistically, miR-3613-5p targeted PTEN, a key tumor suppressor implicated in regulating drug resistance, further emphasizing its potential role in the development of chemoresistance^[109]^.

### Other EV-associated non-coding RNA transfer

Long non-coding RNAs (lncRNAs) have been identified as possible players in the development of chemoresistance, particularly through their association with EVs. By altering gene expression, lncRNAs can influence the cellular mechanisms responsible for drug resistance, including apoptosis, drug efflux, and EMT. For example, EV-mediated transfer of the lncRNA H19 has been demonstrated to enhance the chemoresistance of breast cancer to DOX^[110]^. After observing that H19 expression was upregulated in DOX-resistant BC cell lines (MCF-7 and MDA-MB-231), treatment of the culture medium with RNase confirmed that H19 was delivered via incorporation into EVs and this EV-mediated transport conferred DOX resistance. Interestingly, serum levels of EV-associated H19 were higher in DOX-insensitive patients compared to those with DOX-sensitive tumors. Tao *et al*.^[111]^ examined the role of lncRNA metastasis-associated lung adenocarcinoma transcript 1 (MALAT1) transferred by BC cell-derived EVs. MALAT1 was highly expressed in cells and their derived EVs, where it was shown to enhance malignant properties and chemoresistance. *In vivo* assays confirmed that EV-MALAT1 promoted tumor growth, previously described through its inhibition of miR-1-3p^[112,113]^. This inhibition resulted in the upregulation of VASP and activation of the Rap1 signaling pathway, which in turn enhances proliferation, invasion, and chemoresistance^[111]^.

Non-coding circular RNAs (circRNAs) have been found to be abundant in EVs and have shown a potential role as biomarkers for cancer diagnosis^[114]^. Particularly, circBACH1 has been observed to promote hepatocellular carcinoma and colorectal cancer progression^[115,116]^. The effects of EV-associated circBACH1 on the progression of BC and its potential role in regulating chemoresistance and metastasis were investigated for the first time by Xia *et al.*^[117]^. They demonstrated that circBACH1 was highly expressed in EVs derived from paclitaxel-treated BC cells.

These paclitaxel-induced EVs, containing circBACH1, promoted stemness and migration, while also reducing sensitivity to treatment in breast cancer cells through the miR-217/G3BP2 axis. The lnc RNA OPA-interacting protein 5 antisense transcript 1 (OIP5-AS1) was found to be significantly upregulated in BC^[118]^. Yu *et al*. analyzed EV-mediated trastuzumab (TR) resistance, showing that OIP5-AS1 was upregulated in TR-resistant cells (SKBR3-TR and BT474-TR cells), and this knockdown restored TR sensitivity^[119]^. EVs enriched with OIP5-AS1 from these cells were internalized by TR-sensitive cells and spread resistance, inhibiting TR-induced cytotoxicity and tumor growth *in vivo*. In serum samples from 57 BC patients treated with trastuzumab, elevated EV OIP5-AS1 levels were linked to therapy resistance. The authors suggested that the mechanism involved miR-381-3p, which upregulates HMGB3 expression. MiR-381-3p, an anti-tumor miRNA, suppresses cell proliferation, cell cycle progression, and migration in breast cancer^[120]^, while HMGB3 silencing inhibits cell growth and progression in breast cancer^[121,122]^.

### Non-genetic EV-mediated mechanisms involved in chemoresistance

Despite the great amount of works investigating the role of specific EV-delivered miRNAs in spreading a chemoresistant phenotype to drug-sensitive cells, some authors focused on analyzing other possible EV-related mechanisms underlying the same observed effect. In 2014, Lv *et al*.^[123]^ individuated the delivery of EV-enriched in P-glycoprotein (P-gp) as a means of transferring drug resistance in MCF-7 BC cells. P-gp is an ATP-dependent transmembrane protein that can control the efflux of drug substrates to regulate intracellular levels. Its overexpression is a known mechanism of drug resistance^[124,125]^.

In 2017, Yang *et al*.^[126]^ evaluated the possible role of glutathione S-transferase P1-containing EVs (GSTP1-EVs) in transferring drug resistance to adriamycin. An increase in GSTP1 expression levels was found in drug-resistant cells and their corresponding EVs. In addition, these EVs were able to transmit drug resistance to sensitive cells whose GSTP1 expression was consequently found upregulated. A clinically relevant observation also came from the same study after analyzing the circulating EVs (serum) from 30 patients treated with anthracycline/taxane-based chemotherapy. The expression of GSTP1 in EVs derived from the serum of chemotherapy-resistant patients was significantly higher compared to that of patients who responded to the treatment. More recently, an alternative possible mechanism of adriamycin resistance was proposed by Hu *et al*.^[127]^, demonstrating that small EVs derived from resistant BC cells could transmit drug resistance to sensitive cells by delivering Hsp70 protein. The contribution of EV-delivered Hsp70 to adriamycin resistance was also validated in zebrafish. Hsp70 can translocate to mitochondria in recipient cells, disrupting mitochondrial respiration and promoting glycolysis. While normal cells rely on oxidative phosphorylation for energy, cancer cells switch to glycolysis in the hypoxic tumor microenvironment, supporting growth and contributing to therapeutic resistance^[128-130]^. The EVs-mediated transfer of TGF-β was also proposed by Tan *et al*.^[131]^ as a possible mechanism for adriamycin-resistance transmission.

## CONCLUSION

To date, numerous studies have investigated the potential role of extracellular vesicles in mediating chemoresistance in breast cancer, mainly focusing on EV-genetic cargo^[97,100,104-106]^**.** Most of the mechanisms identified and discussed in this review are based on the ability of these nanoparticles to transport genetic material, particularly miRNAs, which can target specific pathways involved in cell proliferation, stemness, apoptosis, and thus, indirectly, drug resistance. Despite the growing body of literature on this subject, the exchange of miRNAs via EVs as a mechanism of chemoresistance in BC remains a highly debated topic in the scientific community and significant challenges persist, including variability in EV isolation protocols and miRNA profiling techniques, which may affect the reproducibility and interpretation of these findings. Beyond miRNAs, other EV cargo molecules have been implicated in chemoresistance, including long non-coding RNAs (lncRNAs), proteins such as P-glycoprotein, and metabolites that modulate tumor metabolism and the tumor microenvironment. While the functional relevance of these molecules has been reported in several studies^[110,117,119,123,127]^, the relative contribution of miRNA-mediated versus non-miRNA-mediated mechanisms to overall chemoresistance remains an open question. Moreover, the heterogeneity of EV populations, the microenvironmental conditions, and drug regimens further complicate the interpretation of these findings. Given these challenges, it is evident that more physiologically relevant experimental models are needed to validate these mechanisms and their clinical implications. Future studies should explore the role of EVs in patient-derived xenograft (PDX) models, which better replicate the complexity of the clinical environment. Such approaches could provide more reliable insights into the contribution of EVs to chemoresistance and guide the development of novel therapeutic strategies. For instance, prior research using xenograft models of breast cancer^[132]^ highlighted the importance of tailored experimental designs in uncovering novel therapeutic strategies. Additionally, advances in technology, such as single-cell analysis, could offer new avenues to study both tumor cells and, consequently, EV heterogeneity and the EV interactions with the tumor microenvironment. Indeed, recent findings, including single-cell immune data^[133,134]^, demonstrated the potential of integrating these approaches to unravel the complexity of EV-mediated communication. Furthermore, the application of emerging tools such as ultrasound for vesicle tracking and modulation could further enhance our understanding and therapeutic exploitation of these nanoparticles^[135]^. All these challenges and the related findings are of crucial importance, especially from a clinical perspective. EVs could hold significant potential as both prognostic and predictive biomarkers for monitoring therapy response in BC^[96,136]^. In addition, they could serve as therapeutic targets to enhance treatment outcomes. Two ongoing clinical trials, initiated in 2021, are evaluating EVs in BC patients post neoadjuvant chemotherapy (NCT05955521; NCT05831397) (ClinicalTrials.gov). These studies aim to provide valuable insights into the dual potential of EVs as biomarkers and therapeutic targets, thereby advancing the clinical translation of EV-based approaches. A promising strategy to prevent the transfer of resistance profiles from therapy-insensitive cells to those still sensitive to treatment could involve the integration of EV inhibitors with conventional chemotherapy regimens^[137]^. By targeting EVs, this approach could potentially disrupt the transfer of resistance factors, thereby enabling the overall effectiveness of chemotherapy and helping to maintain the sensitivity of tumor cells to treatment.

## DECLARATIONS

### Copyright

© The Author(s) 2025.
